# Assessing the effect of seasonal malaria chemoprevention on malaria burden among children under 5 years in Burkina Faso

**DOI:** 10.1186/s12936-022-04172-z

**Published:** 2022-05-06

**Authors:** Fati Kirakoya-Samadoulougou, Vincent De Brouwere, Arnold Fottsoh Fokam, Mady Ouédraogo, Yazoumé Yé

**Affiliations:** 1 Centre de Recherche en Epidémiologie, Biostatistique et Recherche Clinique, Ecole de Santé Publique, Université Libre de Bruxelles (ULB), Route de Lennik, 808, 1070 Brussels, Belgium; 2 Department of Public Health, Institute of Tropical Medicine, Antwerp, Belgium; 3 ICF, Maroua, Cameroon; 4 Institut national de la statistique et de la démographie (INSD), Ouagadougou, Burkina Faso; 5 ICF, 530 Gaither Road, Rockville, MD 20850 USA

**Keywords:** Seasonal malaria chemoprevention, Evaluation, Impact, Malaria, Burkina Faso

## Abstract

**Background:**

In 2014, the Burkina Faso government launched the Seasonal Malaria Chemoprevention (SMC) programme. Expected benefit was a 75% reduction of all malaria episodes and a 75% drop of severe malaria episodes. This study assessed SMC efficiency on malaria morbidity in the country after 2 years of implementation.

**Methods:**

Quasi-experimental design comparing changes in outcomes during the high transmission period (August–November) between SMC and non-SMC health districts before (2013–2014) and after intervention (two rounds in 2015 and 2016). Health indicators (number of uncomplicated malaria cases (UM) and severe malaria cases (SM)) from 19 health districts (8 in intervention and 11 in comparison group) were extracted from the District Health Information System (DHIS2)-based platform including health facilities data. Effect on incidence was assessed by fitting difference-in difference mixed-effects negative binomial regression model at a log scale.

**Results:**

The two rounds of SMC were associated with a reduction of UM incidence (ratio of incidence rate ratio (IRR) 69% (95% CI 55–86%); p = 0.001) and SM incidence (ratio of IRR = 73% (55–95%), p = 0.018) among under five children.

**Conclusion:**

The two rounds of SMC had a significant effect on the reduction of malaria cases in under five children. This additional evidence on the effectiveness of SMC, using routine data, support the need to sustain its implementation and consider expansion to eligible areas not yet covered.

**Supplementary Information:**

The online version contains supplementary material available at 10.1186/s12936-022-04172-z.

## Background

Malaria control has yielded rewarding achievements in reducing the disease burden in past 15 years due to global revived interest and significant increase in funding [1], which led to the scale-up of multiple control interventions in endemic countries. These control interventions include appropriate and prompt management of uncomplicated with effective and severe cases, the provision of insecticide-treated nets (ITN), indoor residual spraying (IRS), intermittent preventive treatment for pregnant women (IPTp) and, lately, seasonal malaria chemoprophylaxis (SMC) [2].

The World Health Organization (WHO) recommended SMC for malaria prevention in children under five living in markedly seasonal malaria transmission areas in the Sahel region for eligible countries in 2012 [3, 4]. The SMC is defined as “the intermittent administration of full treatment courses of an anti-malarial medicine during the malaria season to prevent malarial illness with the objective of maintaining therapeutic anti-malarial drug concentrations in the blood throughout the period of greatest malarial risk” [5]. As of 2019, twelve countries have been implementing SMC in the Sahel subregion [6], including Burkina Faso, which adopted and launched SMC with sulfadoxine-pyrimethamine + amodiaquine (SP-AQ) in 2014 [7], gradually deployed in health districts over a 6-year period until 2019 [8–12], alongside several other ongoing malaria control interventions, and strengthening of health surveillance system to better monitor and measure achievements.

The improved quality of malaria surveillance data provides an opportunity for assessing the impact of SMC country-wide. Most of the experimental studies supporting the recommendation of SMC in children were conducted in West Africa [13–17], but there is limited evidence for its effect in real-life large-scale conditions. Indeed, until recently few impact evaluations were conducted in the Sahel region, and they found results with different magnitude [18–20]. In Burkina Faso, an impact evaluation using point and period prevalence of parasitaemia, prevalence of moderate to severe anaemia, and history of reported fever in last 14 days as outcome measures was carried out and found a protective efficacy of 51% and 62%, respectively. However, this study was conducted in only one health district, and this could have limited its capacity to capture disparities between health districts in which the SMC was later deployed [9]. Recently, Access-SMC partnership [21] assessed the impact of SMC in the reduction of malaria incidence in seven countries including Burkina Faso. However, at the time of their impact evaluation, data on severe malaria cases were not available for the year 2016 in Burkina Faso and only cases in district hospitals attributed to malaria were considered. The 2016 severe malaria and deaths data in primary health facilities and district hospitals are now available in Burkina Faso, therefore, accounting for both cases and deaths in these primary health facilities and all cases in each year could provide more robust evidence of the effect of SMC in the country.

Given the extensive resources involved in SMC it is crucial to assess how well it works to better inform potential adjustments to optimize the impact of the intervention. This study evaluates the effect of the SMC in children under five living in Burkina Faso during two rounds of implementation using data from the routine health information system.

## Methods

### Country profile

Burkina Faso is a West African country covering an area of 274,200 km^2^ with an estimated population of 20,870,060 inhabitants in 2019. The country is located at a transitional zone between the arid Sahara in the north and the Sudanian zone in the south. The start, duration of rains, total number of rainy days are highly variable and defined by three ecoclimatic zones: Sahelian zone in the north, Sudanian zone in the south and Sudano-Sahelian zone in between with a total annual rainfall/average annual temperature of less than 600 mm/29 °C, 900–1200 mm/28 °C and 600–900 mm/27 °C, respectively [22–24]. The transmission of malaria is stable, occurring throughout the year with a peak during the rainy season (June–September).

Regarding the health system structure, there are currently three levels: the central level elaborates health policies, the regional level coordinates the implementation, and the district level is operational with a network of health centres in relation with district or regional hospitals. The doctor-nurse-midwife/population and primary health facility/population ratios were 8 and 0.9 per 10,000 inhabitants in 2018 [25], respectively. In the country, SMC started in 2014 in a few districts, then extended in 2015 and in 2016. In 2019, every district in Burkina Faso implemented SMC [7–12].

### Study design

The present study is a two-group two-period quasi-experiment with a pre- and post-intervention observations in the country health districts. The intervention being SMC in children under five. In Burkina Faso, the SMC intervention consists in the administration of a monthly 3-day course of SP-AQ (a single dose of SP + 3 days-course of AQ) to children under five on a monthly basis in the rainy months (July–October). The SP-AQ doses are administered by community health workers under the supervision of health providers operating the primary health facility [2].

### Selection of SMC and non-SMC health districts

**Fig. 1:**
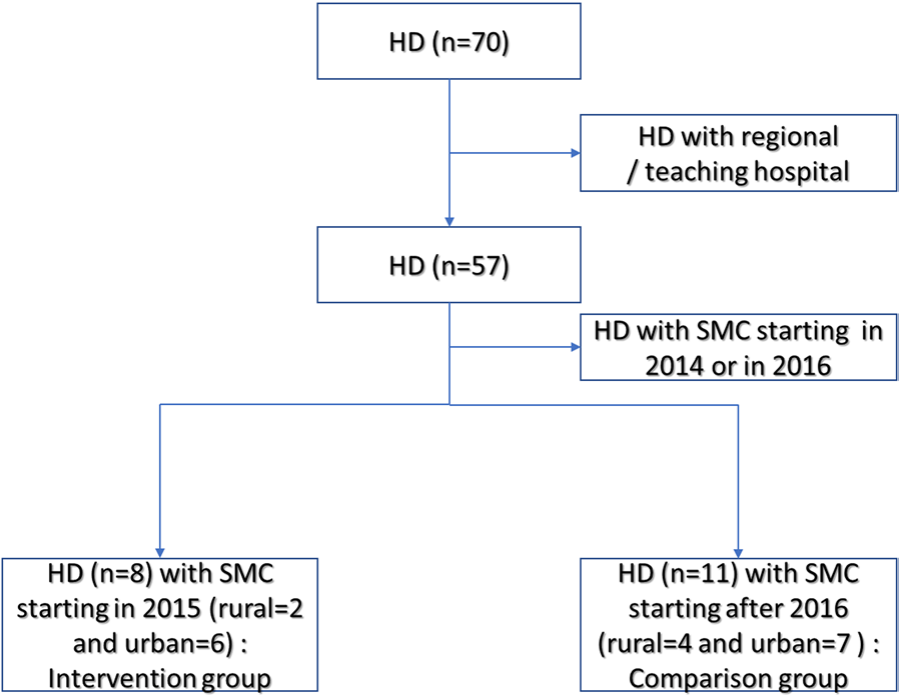
Health district selection flowchart. *HD* health district, *SMC* seasonal malaria chemoprophylaxis

**Fig. 2:**
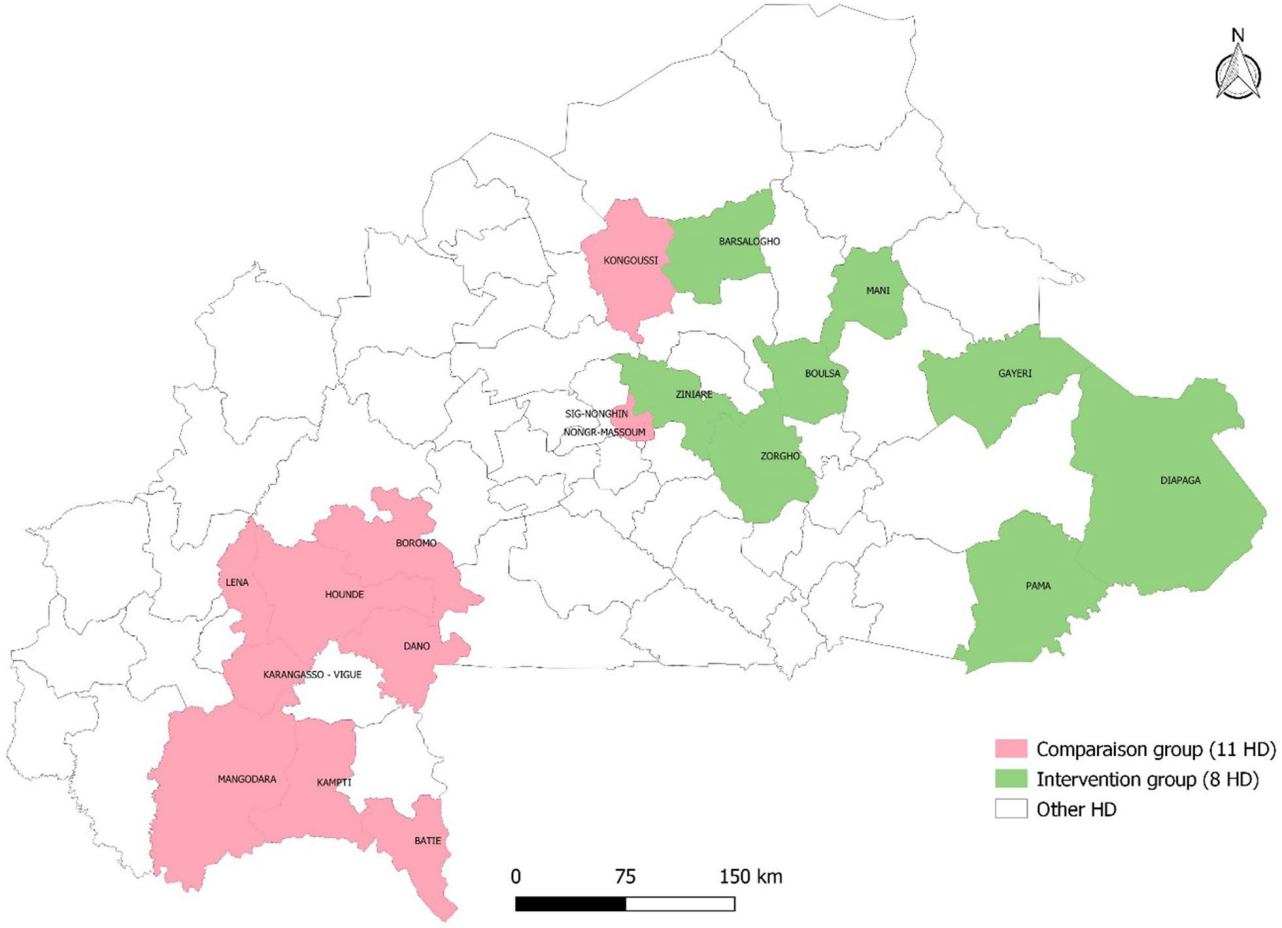
Map of the selected health districts SMC in green and no-SMC in pink

In 2013, the health statistics yearbook listed 70 health districts (primary health facilities and district hospitals data). The inclusion criteria for the intervention group were SMC implemented in 2015 and 2016 [8] and for the comparison group, no SMC before 2017 [7–9]. The exclusion criterion was the presence of a regional or teaching hospital in the district for both groups. Health districts with such hospitals were excluded because their data reported do not discriminate patients from SMC and no SMC districts. Diébougou health district in which IRS was conducted for 3 consecutive years from 2010 was excluded. Indeed, evidence from the Garki project suggests that malaria vector populations, and therefore transmission, may not surge back early after the end of spraying campaigns [6]. In this respect, the Diébougou health district might be different from the other health districts in terms of transmission intensity. The selection process and outcome are summarised in Figs. 1 and 2. Figure 2 presents SMC and non–SMC health districts.

### Data sources

Data were extracted from the online national health data repository which is a District Health Information System (DHIS2)-based platform called ENDOS-BF (*Entrepôt National de Données Sanitaires du Burkina Faso*), operative as of 2013. The monthly data reports on primary health facilities and district hospitals activities sent to the health district, are aggregated in ENDOS-BF.

### Selection of study variables

Among expected benefit of SMC, there was a 75% drop of the incidence of all malaria episodes and a 75% drop of the incidence of severe malaria episodes. The study investigated the effect of the SMC on malaria morbidity based on two outcomes variables collected from primary health facilities and district hospitals to comply with the national policy of malaria case definition: incidence of uncomplicated malaria (UM), i.e. all presumptive cases based on clinical symptoms or confirmed parasitological cases of a malaria rapid diagnostic tests (RDT) or microscopy and incidence of severe malaria cases in children (SM) i.e. confirmed cases of malaria with signs of severe illness and/or evidence of vital organ dysfunction who required hospitalization [7]. The incidence of UM in children under five is defined as the number of UM reported cases, relative to the total population of children under five living in the health district. The incidence of SM in children under five is defined as the number of SM reported cases, relative to the total population of children under five living in the health district. There were also the data for older age groups (5–14 years and 15 years and more) to control for confounding temporal trends.

### Data quality assurance

Data quality audits are conducted periodically in public primary health care facilities by supervisory teams from the district level to check consistency between source data (registers) and data reported in the monthly activities reports. Besides, in parallel to the mandatory global monthly activity reports, each health facility produces a weekly telegram on key health indicators among which uncomplicated and severe malaria cases in children under five. The figures reported to the Health Statistics Directorate of the Ministry of Health should match those of the aggregated weekly telegrams reported to the disease surveillance directorate of the same ministry. ENDOS-BF has built-in quality, which enables central data managers to check several dimensions of quality in the course of data entry and generate queries intended for districts data managers [26]. An assessment of ENDOS-BF in 2017 reported data accuracy, data timeliness, data completeness, and data reliability of 83%, 80%, 78% and 87%, respectively [27]. Moreover, the data discrepancy index between the source and the reported data improved from 61% in 2013 to 80% in 2018 [28].

### Data analysis

#### Background characteristics of health districts in intervention and comparison groups

The study health districts were comparable in many respects at baseline (2013). The differences in surface area is attributable to one significantly wider district (Diapaga) in the intervention group. There was no statistical difference in terms of average annual rainfall and average annual temperature (overlapping of 95% confidence interval). The ratio population per health worker, in the intervention districts is lower compared to comparison districts. The proportion of population living at less than 5 km away from a health facility is also substantially lower in the intervention districts. Globally, bed net ownership was lower in the SMC group compared to non-SMC group in 2014, and bed net use proportion are similar in both groups before SMC implementation (Table 1).

**Table 1:** Summary of study health districts characteristics in 2013

Background characteristics	SMC health districts (intervention)	Non-SMC health districts (comparison)
Total surface area (km^2^)	46,785	33,833
Average annual rainfall (mm) (95% CI)	794 (640, 948)	752 (629, 876)
Average monthly temperature (°C) (95% CI)	28.1 (27.7, 28.6)	28.0 (27.6, 28.4)
Total population	2,042,236	2,133,367
Total number of primary health facilities	212	199
Total health staff	1150	2173
Population/health facility ratio	9633	10,720
Population/health care worker ratio	1776	981
Population of children under five		
Total	422,272	382,191
Proportion	20.7%	17.9%
Population at < 5 km away from the health facility		
Total	808,066	1,363,340
Proportion	39.6%	63.9%
Insecticide treated nets in 2014^a^		
Ownership	75.4%	82.2%
Use	88.0%	87.2%

^a^We obtained this information from the Malaria Indicator Survey (MIS) conducted in 2014. The MIS was not designed to provide estimates at health districts level. To address this issue, we fitted a Bayesian binomial model to the aggregated data on ITN ownership and use to estimate them at the district level

#### Statistical analysis (descriptive and regression)

For each outcome (uncomplicated malaria incidence and severe malaria incidence) and each group, the average difference between the post-(combining the data of first round August 1 to November 30, 2015, and second round August 1 to November 30, 2016) and pre-(August 1 to November 30, 2013, and August 1 to November 30, 2014) intervention periods were calculated as previously done [21]. The average change was then subtracted in the outcomes over time in health districts not implementing SMC from that of health districts implementing the SMC to single out effect of SMC. The parallel trend assumption guides the use of the difference in difference (DID) analysis and assumes that no time-varying differences exist between the SMC and comparison groups. For that purpose, a test was performed verifying whether variations in outcomes were equivalent between the two groups during the pre-intervention period. Due to overdispersion in the data, a mixed-effects negative binomial regression model was fitted. Given the geographic variation in malaria incidence, heath districts were included as random intercepts.

In 2016, Burkina Faso removed patient charges for children under 5 years and for pregnant women. This was associated with a marked increase in outpatient attendance and hence a marked increase in reported case of malaria. For that, the authors considered the variable as a control variable in the difference-in-difference analysis to estimate the impact of SMC accurately.

The validity of the regression was included by comparing Akaike Information Criterion (AIC) of Poisson regression and binomial negative Regression. With the lowest AIC, a mixed-effects negative binomial regression model was fitted to estimate the effect of SMC on incidence of uncomplicated malaria cases, and severe malaria cases. Each regression model included time variable (pre-SMC period vs post-SMC period), SMC variable (SMG group vs non-SMC group, the free healthcare variable (Free_Healthcare) and population size (children under five aged in each district) as an offset.

The empirical model estimated can be characterized as:

$$ Y_{it} = \beta_{0i} + \beta_{1} Time + \beta_{2} SMC + \beta_{3} Free\_Healthcare + \beta_{4} SMC*Time + \varepsilon_{it} $$

where *Y*_*it*_ is number of cases of interest provided by district *i* in time period *t* (*in months*), time is a variable capturing the pre (2013–2014) and post-SMC period (2015 and 2016), SMC is an indicator for a health district being in a SMC group or non-SMC group, Free_healthcare variable for patient removing charges, SMC * Time the interaction that captures the DID estimator at log scale, the effect of the implementation of two rounds of SMC in 2015 and 2016 and $\varepsilon_{it}$ the error term.

From the mixed-effects negative binomial regression models, ratio of incidence, rate ratio with 95% confidence interval and p-values were estimated. Statistical significance was met for p-values < 0.05. All analyses were performed using Stata (version 16; StataCorp, College Station, TX, USA).

## Results

### Crude trends in uncomplicated malaria and severe malaria monthly incidence

**Fig. 3:**
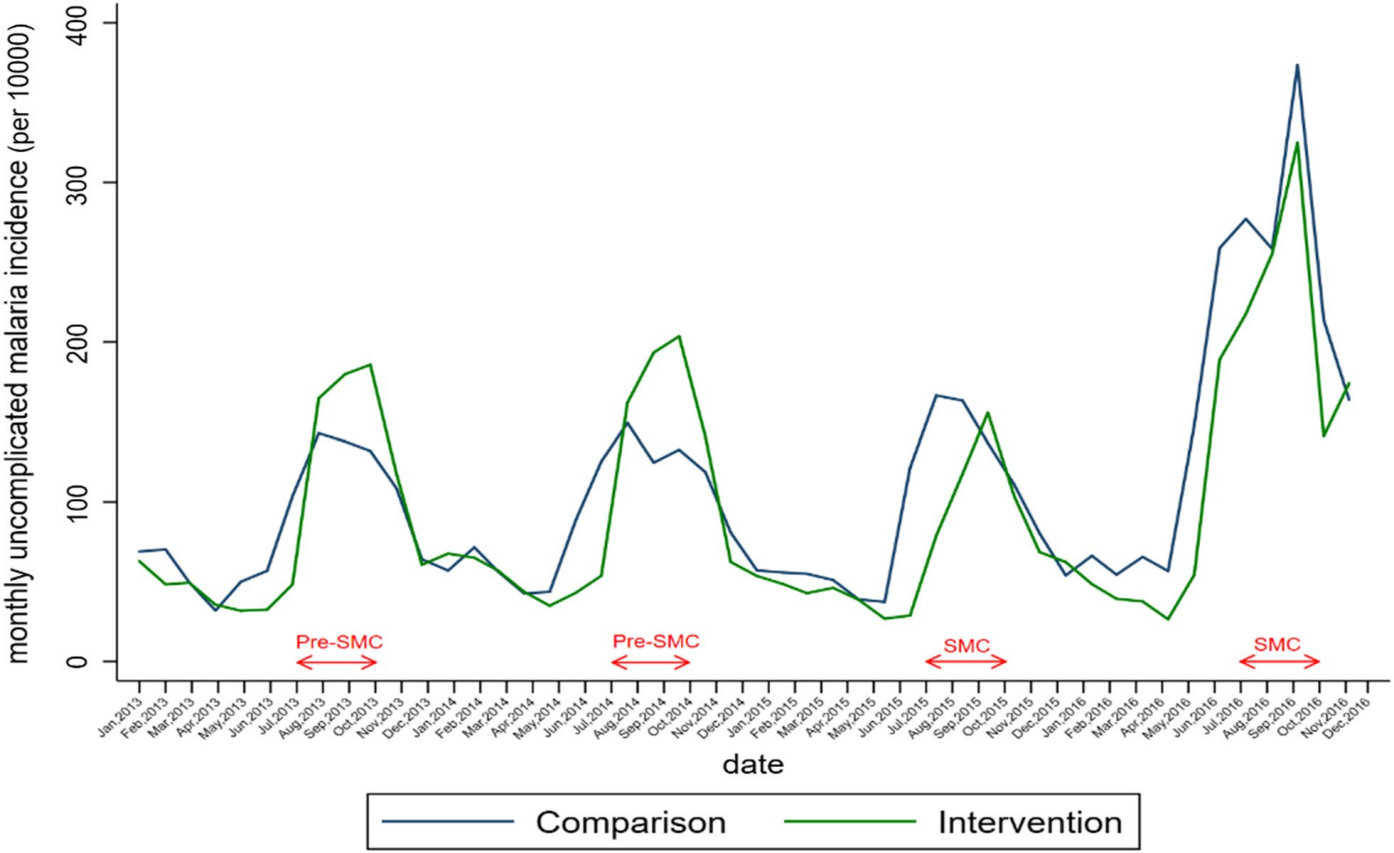
Monthly trends of uncomplicated malaria incidence in health districts before and after SMC implementation

**Fig. 4:**
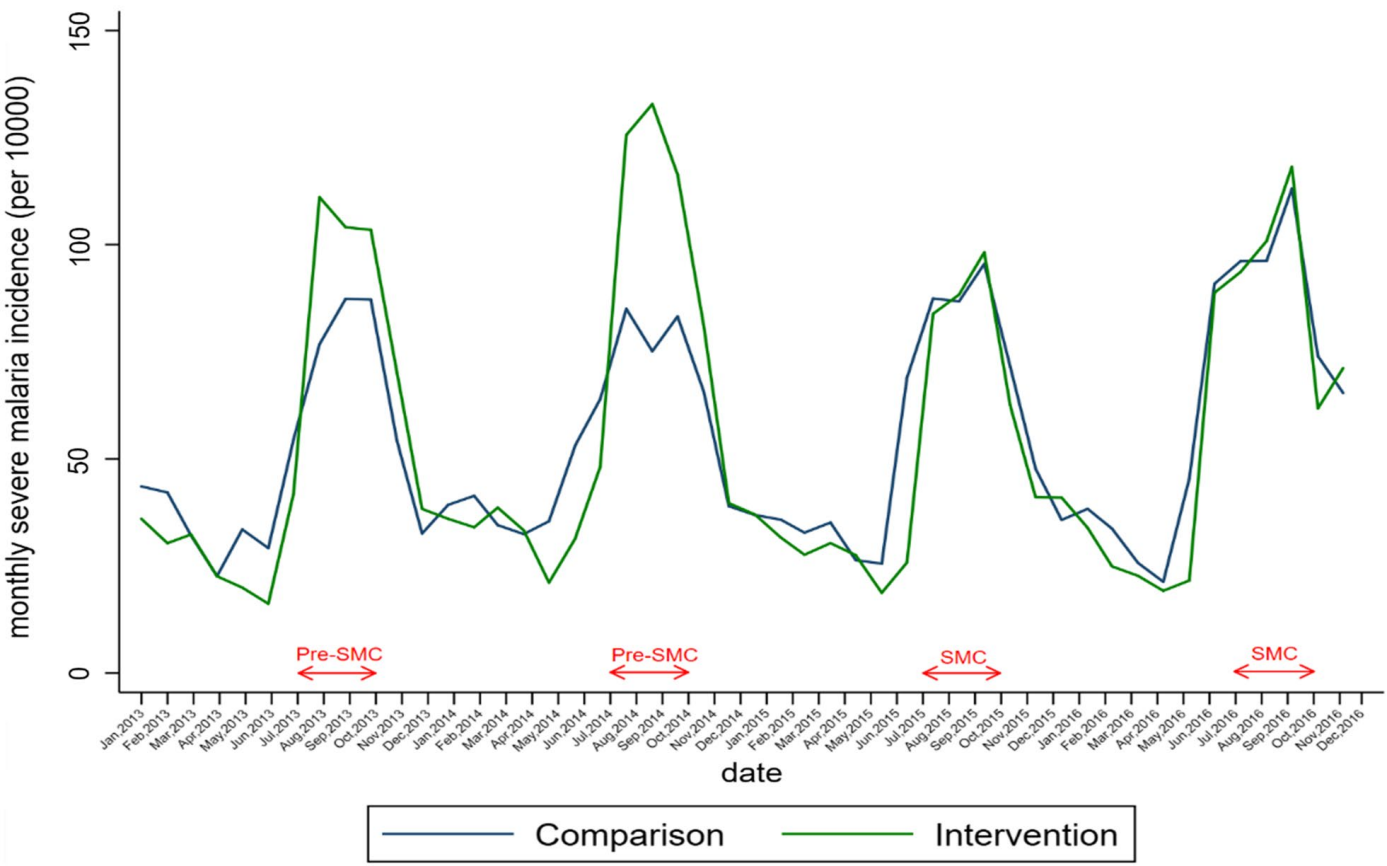
Monthly trends of severe malaria incidence in health districts before and after SMC implementation

Table 2, Figs. 3 and 4 present the crude trends of uncomplicated malaria and severe malaria monthly incidence respectively. Before the introduction of SMC, the crude monthly uncomplicated malaria incidence during high transmission period was higher in SMC group decreased after the two rounds of SMC to the level of the comparison group (Table2, Fig. 3). Before the establishment of SMC, the SMC group showed a high evolution of crude monthly severe malaria incidence during the high transmission period to that of control. Following the first and the second round of SMC implementation, there was a decline in the SMC group. (Table 2, Fig. 4).

**Table 2:** Number of uncomplicated and severe malaria cases, testing rate by group and year (August–November) among children under 5 years, 2013–2016

	2013	2014	2015	2016
Uncomplicated malaria cases				
Comparison group	209,816	216,047	244,386	487,104
SMC group	264,905	293,816	196,534	387,255
Severe malaria cases				
Comparison group	13,071	13,244	14,796	15,975
SMC group	14,887	17,483	12,746	14,360
Malaria suspected cases				
Comparison group	224,102	239,942	305,405	473,295
SMC group	279,499	311,299	209,756	364,534
Suspected malaria cases tested				
Comparison group	127,912	184,858	249,880	485,624
SMC group	166,575	259,008	219,637	351,972
Malaria confirmed cases				
Comparison group	115,702	168,142	221,919	394,583
SMC group	155,441	239,876	197,776	279,676
Testing rates (%)				
Comparison group	57.1	77.0	81.8	102.6
SMC group	59.6	83.2	104.7	96.6
Test positivity rate (%)				
Comparison group	90.5	91.0	88.8	81.3
SMC group	93.3	92.6	90.0	79.5

The crude trends in uncomplicated malaria and severe malaria monthly incidence for older age group was presented in Additional file 1: Figs. S1, S2. Malaria testing rates was higher in the SMC group compared to non-SMC group between 2013 and 2015, even in 2016 the proportion was higher in the comparison group compared to SMC group. However, the test positivity rate was similar for both groups during the study period 2013–2016 (Table 2).

### Effect of seasonal malaria chemoprevention on malaria burden

Compared to the period before implementation and after the two rounds of SMC, incidence rates of UM increased in the comparison group [incidence rates ratio (IRR): 1.05 (95% CI 0.92–1.20)] while in the SMC group, the incidence rate was significantly lower [IRR: 0.72 (95% CI 0.63–0.82)]. The ratio of the IRR in SMC and comparison group [69% (95% CI 55–86%; p = 0.001)] reflects an important difference in the evolution of UM incidence rates between the two groups, in favour of the SMC group (Table 3). Compared to the period before implementation and that after the two rounds of SMC, incidence rates of SM increased in the comparison group [incidence rates ratio: 1.08 (95% CI 0.93–1.27)] while in the SMC group, the incidence rate was significantly lower [IRR: 0.79 (95% CI 0.68–0.91) 0.64–0.95)]. The ratio of the IRRs in SMC and comparison group [73% (95% CI 55–95%, p = 0.018)] reflects an important difference in the evolution of SM incidence rates between the two groups, in favour of the SMC group (Table3). The positive impact of SMC among children under five was not observed among older age group (Additional file 1: Tables S1, S2).

**Table 3:** Effect of seasonal malaria chemoprevention on incidence of uncomplicated and severe malaria cases

Period	Mean incidence rates (100 persons-months)		Comparison	Intervention	IRR^a^ ratios (%) (95% CI)	p
	Comparison districts (n = 11)	Intervention districts (n = 8)	IRR (95% CI)	IRR (95% CI)		
Uncomplicated malaria						
Before	12.7	15.0	1	1	1	
After	13.2	10.7	1.05 (0.92–1.20)	0.72 (0.63–0.82)	69 (55–86)	0.001
Severe malaria						
Before	7.0	8.9	1	1	1	
After	7.5	7.0	1.08 (0.93–1.27)	0.79 (0.68–0.91)	73 (55–95)	0.018

*IRR* incidence rate ratio obtained from a mixed-effects negative binomial regression model with random intercept for health districts and robust standard error

^a^Ratio of IRR intervention vs. IRR comparison

## Discussion

This study assessed the effect of seasonal malaria chemoprevention in children under five on malaria morbidity using routine data in Burkina Faso from 2013 to 2016. After two rounds of SMC, we observed a significant decrease in the incidence of uncomplicated malaria cases and severe malaria cases.

These findings are in line with the well-documented effectiveness of SMC in reducing severe malaria incidence. In a quasi-experimental study based on one comparison district (Bafoulabe) and one intervention district in Kita, Mali, Diawara et al. found that the SMC reduced severe malaria incidence by 72% [18] in 2014 compared to 2013. A previous study conducted in one district (Kaya, Burkina Faso) at the early stage of the SMC implementation in the country showed that it could reduce febrile episodes by 46% but did not specifically investigate its effect on severe malaria [19]. The results also confirm the findings of ACCESS SMC on a large scale in Burkina Faso. By also using routine data in Burkina Faso, the associated reduction in SM cases with SMC estimated from Poisson regression was 27.4% in 2015 [21].

Moreover, a large-scale randomized trial published in 2021, in which the efficacy of SMC was estimated by comparing malaria incidence in children who received SMC plus the malaria vaccine with children who receive only the malaria vaccine [29]; and a re-analysis of large scale randomized trial of SMC combined with azithromycin [30] have shown results, are consistent with a high level of efficacy of SMC.

This study also observed an increase in malaria incidence in 2016. This calls for the exploration of new strategy like a combination of SMC with malaria vaccine, strengthening SP resistance monitoring, extend age group of target population, expending the period to at least 3 months after the high transmission period, active surveillance at the community level. A positive contribution of combination of malaria vaccine and SMC on malaria burden has been reported recently [31].

This study demonstrates the valued added of using routine data to effectively monitor and evaluate the effect on malaria interventions at the subnational level. This calls for a need to continuing strengthening routine information systems for improved quality of malaria surveillance data and use of these data to inform program implementation. In line with the High Burden High Impact Strategy (HBHI) regular subnational assessment of change in malaria burden, is necessary for defining appropriate intervention package targeted to specific area.

## Limitations

There are a number of limitations to this study. The relative contribution of other ongoing interventions that could have an effect on malaria morbidity (e.g. ACT for effective treatment, use of ITNs and proper management of malaria cases) and SMC coverage was not assessed. Therefore, these results cannot be attributed solely to SMC because it is an additional intervention to others, such as IPTp with SP, the use of ITN (ITN. mass distribution campaign and routine distribution) and proper management of malaria cases in the selected area. Moreover, overdiagnosis is likely to be a feature of most programmatic data because diagnosis of malaria in the routine system relies on the use of RDTs. However, even if this burden were to be discounted to account for potential overdiagnosis, it would remain substantial.

Variation in the implementation of strategy between rounds and/or districts can occur and limit the external validity. According to research, repeated cycles of SMC could contribute to reduce overall malaria transmission [32]. In this study it was impossible to investigate this hypothesis because this only focused on the use of routine data to assess SMC impact. Also, the proportion of malaria cases (uncomplicated and severe) not captured by ENDOS-BF and exclusion of districts with regional or teaching hospitals can limit the SMC impact. Due to their attractiveness, they might have concentrated cases of severe malaria from selected districts, which do not have a regional or teaching hospital. Thus, this study can be insufficiently powered to detect SMC impact, especially on severe malaria.

However, despite its limitations, the authors have mostly looked at SMC impact from the data perspective because other context issues related SMC are already extensively documented in the literature. SMC is an effective strategy if well implemented with effective drug, perfect timing, and optimal coverage. Moreover, SMC in mainly for burden reduction, not elimination strategy. It is then critical to put in place a function data system to monitor SMC and measure its impact.

## Conclusions

The two rounds of SMC implementation were associated with a reduction in uncomplicated and severe malaria cases among children under 5 years. This additional evidence on the effectiveness of SMC, using routine data, support the need to sustain its implementation and consider expansion to eligible areas not yet covered. There is also a need to continue efforts in strengthening routine health information system to provide quality data for monitor coverage and assess impact on malaria burden at the subnational level.

## Supplementary Information

**Additional file 1: Figure S1.** Monthly trends of uncomplicated malaria incidence in health districts before and after SMC implementation among older age group (5–14 years old and 15 years and more). **Figure S2.** Monthly trends of severe malaria incidence in health districts before and after SMC implementation among older age group (5–14 years old and 15 years and more). **Table S1.** Effect of seasonal malaria chemoprevention on incidence of uncomplicated malaria cases among older age group (5–14 years old and 15 years and more). **Table S2.** Effect of seasonal malaria chemoprevention on incidence of severe malaria cases among older age group (5–14 years old and 15 years and more).

## Data Availability

All data were extracted from the national health information system as part of routine care. Data that support the findings of this study are available at the Ministry of Health in Burkina Faso.

## Abbreviations

AIC: Akaike Information Criterion
CI: Confidence interval
CNRFP: Centre National de Recherche et de Formation sur le Paludisme
DHIS: District Health Information System
DID: Difference in Difference
ENDOS-BF: Entrepôt National de Données Sanitaires du Burkina Faso
INSD: Institut national de la statistique et de la démographie
IPTp: Intermittent Preventive Treatment for pregnant women
IRR: Incidence rate ratio
ITN: Insecticides-treated bed nets
LLIN: Long-lasting insecticidal nets
RDT: Rapid diagnosis test
SMC: Seasonal malaria chemoprevention
SMC: Severe malaria
SP–AQ: Sulfadoxine–pyrimethamine + amodiaquine
ULB: Université libre de Bruxelles
UM: Uncomplicated malaria
WHO: World Health Organization
