# Supplementary material for: Assessing the effect of seasonal malaria chemoprevention on malaria burden among children under 5 years in Burkina Faso
Source: Malar J. 2022 May 6;21:143. doi: 10.1186/s12936-022-04172-z (PMC9074217; doi:10.1186/s12936-022-04172-z)
Supplement: Supplementary file 1 — Additional file 1: Figure S1. Monthly trends of uncomplicated malaria incidence in health districts before and after SMC implementation among older age group (5–14 years old and 15 years and more). Figure S2. Monthly trends of severe malaria incidence in health districts before and after SMC implementation among older age group (5–14 years old and 15 years and more). Table S1. Effect of seasonal malaria chemoprevention on incidence of uncomplicated malaria cases among older age group (5–14 years old and 15 years and more). Table S2. Effect of seasonal malaria chemoprevention on incidence of severe malaria cases among older age group (5–14 years old and 15 years and more). [file 12936_2022_4172_MOESM1_ESM.docx]

**Additional file 1**

**
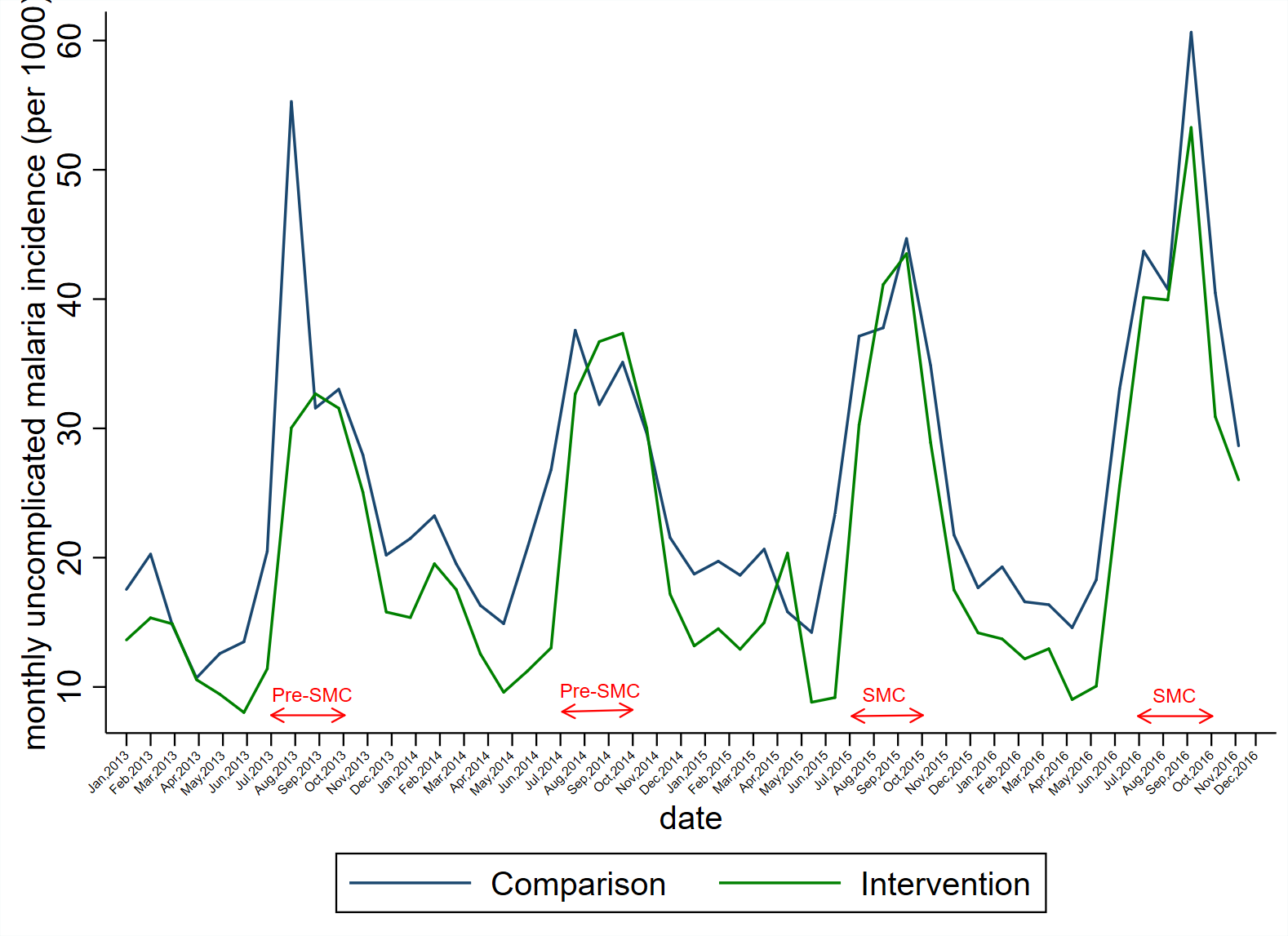
**

Figure S1. Monthly trends of uncomplicated malaria incidence in health districts before and after SMC implementation among older age group (5-14 years old and 15 years and more).


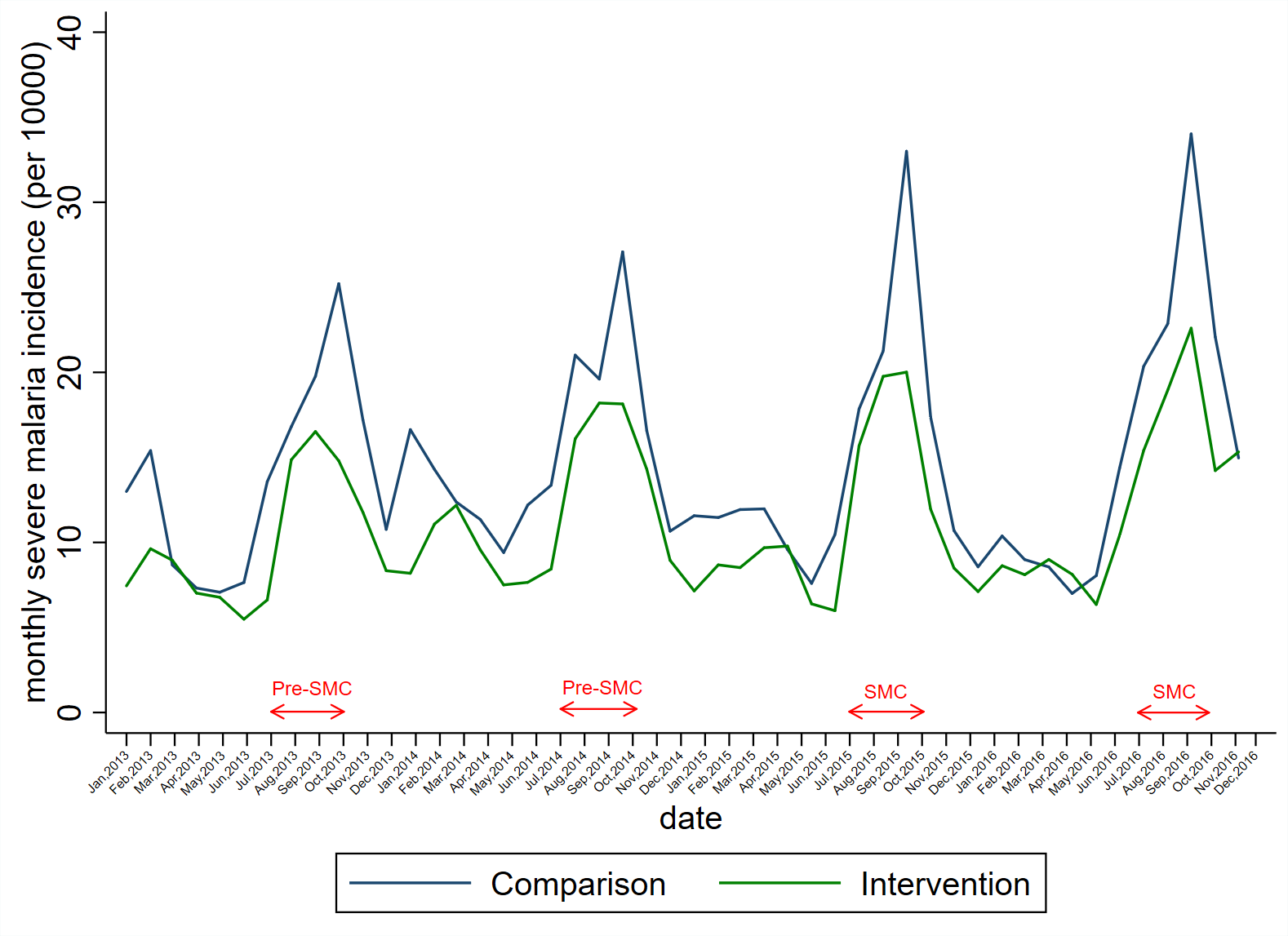


Figure S2. Monthly trends of severe malaria incidence in health districts before and after SMC implementation among older age group (5-14 years old and 15 years and more).

**Table S1. Effect of seasonal malaria chemoprevention on incidence of uncomplicated malaria cases** **among older age group (5-14 years old and 15 years and more).**

|  | Mean Incidence rates  (10000 persons-months) | | Control | Intervention |  |  |
| --- | --- | --- | --- | --- | --- | --- |
|  | Conparison districts (n=11) | Intervention districts  (n=8) | IRR (95%CI) | IRR (95% CI) | IRR^a^ ratios (%) (95% CI) | p |
| Period  Before  After | 1.69  1.70 | 1.17  1.20 | 1  1.01 (0.92-1.10) | 1  1.03 (0.91-1.16) | 1  102 (89-117) | 0.79 |

**Table S2. Effect of seasonal malaria chemoprevention on incidence of severe malaria cases** **among older age group (5-14 years old and 15 years and more).**

|  | Mean Incidence rates  (100000 persons-months) | | Control | Intervention |  |  |
| --- | --- | --- | --- | --- | --- | --- |
|  | Comparison districts (n=11) | Intervention districts  (n=8) | IRR (95%CI) | IRR (95% CI) | IRR^a^ ratios (%) (95% CI) | p |
| Period  Before  2015 | 8.79  7.90 | 6.13  5.84 | 1  0.90 (0.79-1.02) | 1  0.95 (0.83-1.09) | 1  106 (88-128) | 0.55 |
